# Supplementary material for: Cultural and contextual adaptation of the Solastalgia subscale of the Environmental Distress Scale in drought-affected Kilifi, Kenya
Source: Glob Ment Health (Camb). 2025 Jan 22;12:e13. doi: 10.1017/gmh.2025.8 (PMC11810755; doi:10.1017/gmh.2025.8)
Supplement: Wahid et al. supplementary material [file S2054425125000081sup001.docx]

Annex-1: Key findings from expert review, focus group discussions and cognitive interviews about the Environmental Distress Scale items in English and Swahili.

| EDS Scale items & initial translation | Finding from Experts | Findings from FGDs | Findings from cognitive interview | Comprehensibility | Acceptability | Relevance | Completeness |
| --- | --- | --- | --- | --- | --- | --- | --- |
| EDS Item-1: My sense of belonging to this place has been undermined by unwelcome change  Initial translation: Hisia yangu ya kuwa wa eneo hili imedhoofishwa na mabadiliko yasiyokubalika | Two experts felt the word 'imedhoofishwa' as a translation of ‘undermined’ was too technical. Recommended replacement with the word 'imedhalilishwa' which is commonly used to convey undermined. | The translated word ‘imedhoofishwa' for ‘undermined’ was found to be too technical. Recommended to replace with a simpler word.  Respondents recommended replacing ‘Yasiyokubalika’ which means ‘unwelcome’ in English to ‘yasiyo ya kawaida’ which translates to ‘unusual things.’ | Participants agreed with the changes recommended by experts and in FGDs. No further changes were recommended.  After recommended changes were made, the statement was found to be comprehensible during cognitive interviews | Initial translation was found to not be comprehensible by all participants.  The updated version was found to be comprehensible by cognitive interview participants after changes identified in FGDs and expert review were incorporated. | Acceptable by all participants | Relevant to the local context | Complete |
| EDS Item-2: I am sad that familiar aspects of this place are disappearing (e.g., animals, plants, landmarks, open space).  Initial translation: Nina huzuni kwamba vipengele vinavyojulikana vya mahali hapa vinatoweka (k.m., wanyama, mimea, alama za jiographia, nafasi za wazi). | The word ‘vipengele’ was identified as too technical. Recommended to change ‘vipengele’ which means ‘aspects’ to ‘maandhari’ which translates to 'nature/environment' to fit the contextual meaning of the Scale. ‘Vipengele’ when back translated to English would have a meaning closer to ‘indicators’, which holds a completely different meaning from the original word EDS construct of ‘aspects’. | FGD 1&2 discussants further recommended to replace the word ‘vipengele,’ which means ‘aspects,’ with a simpler word, ‘vitu,’ which when translated to English would translate to ‘things.’  Also, replacing the word ‘vinatoweka’ was recommended. This word in English stands for ‘disappearing’ and has the implication that such things which have disappeared can be found if searched for, which is not aligned with the EDS item intentionality. The word ‘vimeisha' which more clearly implies 'extinction' was recommended as a replacement. | Participants agreed with the changes recommended by experts and in FGDs. No further changes were recommended. | Not comprehensible according to experts due to the use of the word 'vipengele'.  Not comprehensible to FGD participants because of the words 'vipengele' and 'vinatoweka.  After recommended changes were made, the statement was found to be comprehensible to participants who took part in the cognitive interviews | Acceptable by all participants | Relevant to the local context | Complete with examples provided |
| EDS Item-3: I am worried that aspects of this area that I value are being lost.  Initial translation: Nina wasiwasi kuwa vipengele vya eneo hili ambavyo ninathamini vinapotea | One Expert recommended replacing the word 'vipengele' which translates to 'aspects' to 'vitu' which translates to 'things' for comprehensibility. | Similar to the feedback from expert review, FGD 1&2 discussants recommended replacing 'vipengele' with 'vitu' and provide specific examples to specify what aspects are being talked about. | Participants agreed with the changes recommended by experts and in FGDs. No further changes were recommended. | Not comprehensible to FGD participants and experts. After recommended changes were made, and relevant examples added (rivers, animals, forests, coconut trees), the statement was comprehensible to participants during cognitive interviews, and considered complete | Acceptable by all participants | Relevant to the local context after inclusion of contextually salient examples. | Complete with the added examples. |
|  |  |  |  |  |  |  |  |
| EDS Item-4: I miss having the peaceful feeling that I once enjoyed by being in this place.  Initial translation: Ninakosa kuwa na hisia ya amani ambayo hapo awali nilifurahia kwa kuwa mahali hapa. | No recommended changes | FGD participants felt ‘mahali hapa’ which translates to 'this place' may imply a small restricted area currently inhabited by the respondent and recommended using ‘eneo hili' which signifies a larger geographical area, more salient to what the scale attempts to elicit. The translation used for cognitive interviews was further refined to ‘mwenyeji wa eneo hili’ which means ‘a resident of this place’ providing further context.  FGD participants also recommended replacing 'awali' which translates to 'before' with 'hapo mwanzo' which means 'in the beginning' for cultural saliency reasons. | Participants agreed with the changes recommended by experts and in FGDs. No further changes were recommended. | Comprehensible to experts and cognitive Interview participants. The initial translation was not comprehensible to FGD discussants who recommended several changes. | Acceptable by all participants | Relevant to the local context | Complete |
| EDS Item-5: I am upset at the way this area looks now  Initial translation: Nimekasirishwa na jinsi eneo hili linavyoonekana kwa sasa. | Two experts recommended replacing ‘Nimekasirishwa’ which translates to ‘upset’ with ‘sijafurahia/furahishwa’ which translates to 'unhappy,' but sounds more polite in Swahili. | Similar to expert review recommendations, FGD participants from both FGDs recommended the use of a more polite word to convey ‘upset’ and replace 'nimekasirishwa' with 'sijafurahishwa' | Participants agreed with the changes recommended by experts and in FGDs. No further changes were recommended. | Initial statements were found not comprehensible by FGD participants and experts based on the term 'kukasirishwa'. After recommended changes, the statement was said to be comprehensible to cognitive interview participants | Not acceptable as the word' nimekasirishwa' is impolite and strong and cannot be used to connect to being upset about the environment. With the recommended changes, the statement was found to be acceptable. | Relevant | Complete |
| EDS Item-6: My lifestyle is being threatened by change in my local area  Initial translation: Mtindo wangu wa maisha unatishiwa na mabadiliko katika eneo langu. | Two experts recommended replacing the word 'mtindo' which was used for ‘lifestyle’ with 'hali yangu ya maisha' which translates to ‘my way of life’. One expert recommended making the statement general; that is replace 'unatishiwa’ which in English stands for ‘threatened’ to 'inatishiwa' which makes it more general. | Similar to exper review recommendations, FGD discussants recommended replacing the word ‘mtindo’ with ‘Hali’ which is closer to 'way of life.' Mtindo is generally used to demonstrate things such as fashion, eating patterns etc. Replace 'eneo langu' which means my area' with 'eneo hili' to mean local area | Participants agreed with the changes recommended by experts and in FGDs. No further changes were recommended. | Initial statements were found not comprehensible by FGD participants and experts. Comprehensible to Cognitive Interview participants after recommended changes were made. | Not acceptable to experts and FGD discussants because of the word 'mtindo' which has an unrelated meaning. Once changes were made, the statement was found acceptable to cognitive interview participants | Relevant | Complete |
| EDS Item-7: Unique aspects of nature that made this place special are being lost forever  Initial translation: Vipengele vya kipekee vya asili vilivyofanya mahali hapa kuwa maalum vinapotea milele | Two experts recommended Replacing ‘vipengele’ which in English translates to ‘aspects’ with ‘vitu vya dhamani’ which translates to English to mean ‘valuable things.’  One expert mentioned that the word ‘asili’ which translates to ‘nature’ in English, can be quite limiting to only traditional things. | FGD 2 discussants recommended Replacing ‘vipengele’ which in English translated to ‘aspects’ to ‘vitu’ which would translate to ‘things’ in English.  Replaced 'vipengele' with 'vitu' which means 'things.''Asili' has been replaced with 'Maandhari' to mean nature (asili is limited to traditional things). Recommended removing the word ‘forever’ as some things e.g., plants may grow back once the weather patterns change. | Participants agreed with the changes recommended by experts and in FGDs. No further changes were recommended. | Comprehensible | Acceptable by all participants | Contextually relevant | Incomplete without examples. Found to be complete with addition of locally salient examples by cognitive interview participants. |
| EDS Item-8: I am saddened by unwelcomed change I see in my landscape  Initial translation: Nimehuzunishwa na mabadiliko yasiyokubalika ninayoyaona katika mazingira yangu. | Experts did not recommend any changes | FGD 2 recommended replacing ‘Huzuni’ which in English translates to ‘saddened’ with ‘Kusikitika’ which is synonymous with ‘huzuni’ but fits well in a climate change context. ‘Huzuni’ is linked to severe outcomes such as death.  FGD 1 recommended replacing 'yasiyokubalika’ which in English translates to ‘unacceptable’ with ‘yasiyo ya kawaida’ to convey ‘unusual’ which is better suited for the context. | No recommended changes from cognitive interviews | Comprehensible | Acceptable by all participants | Not relevant because the word 'huzunishwa' is not contextually appropriate. With recommended changes, the statement was found to be relevant | Complete |
| EDS Item-9: I feel powerless to stop unwanted changes to this place  Initial translation: Ninahisi kutokuwa na uwezo wa kukomesha mabadiliko yasiyotakikana mahali hapa. | One expert recommended replacing ‘Yasiyo takikana’ which in English translates to ‘unwanted’ with ‘yasiyo ya kawaida’ which in English would mean ‘unusual.’ | No recommended changes | No recommended changes from cognitive interviews | Comprehensible | Acceptable by all participants | Relevant to the local context | Complete |
